# A unified Gaussian copula methodology for spatial regression analysis

**DOI:** 10.1038/s41598-022-20171-1

**Published:** 2022-09-23

**Authors:** John Hughes

**Affiliations:** Lehigh University, Bethlehem, PA 18015 USA

**Keywords:** Statistics, Gastrointestinal cancer, Computational science

## Abstract

Spatially referenced data arise in many fields, including imaging, ecology, public health, and marketing. Although principled smoothing or interpolation is paramount for many practitioners, regression, too, can be an important (or even the only or most important) goal of a spatial analysis. When doing spatial regression it is crucial to accommodate spatial variation in the response variable that cannot be explained by the spatially patterned explanatory variables included in the model. Failure to model both sources of spatial dependence—regression and extra-regression, if you will—can lead to erroneous inference for the regression coefficients. In this article I highlight an under-appreciated spatial regression model, namely, the spatial Gaussian copula regression model (SGCRM), and describe said model’s advantages. Then I develop an intuitive, unified, and computationally efficient approach to inference for the SGCRM. I demonstrate the efficacy of the proposed methodology by way of an extensive simulation study along with analyses of a well-known dataset from disease mapping.

## Introduction

**Figure 1:**
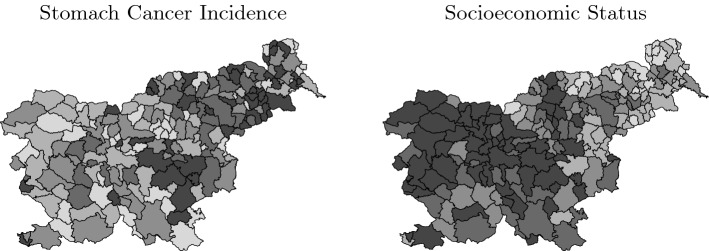
Stomach cancer incidence and socioeconomic status for the municipalities of Slovenia. Figure created using R version 4.1.2 (https://www.r-project.org).

The aim of a spatial regression analysis is to explain a substantial proportion of the spatial pattern exhibited by some dependent variable by appealing to the spatial structure exhibited by one or more independent variables. Consider the data shown in Fig. 1, for example. The response (left panel) is stomach cancer incidence for each of Slovenia’s municipalities for the period 1995–2001^1^. The explanatory variable (right panel) is socioeconomic status. An inverse relationship between the two variables is clearly evident, i.e., higher socioeconomic status (tending toward black) is associated with lower stomach cancer incidence (tending toward white), and vice versa.

Since spatially structured data like the Slovenia data are common in many fields, and explanation, as opposed to interpolation or smoothing, is often desired, spatial regression methods are important data-analytic tools for many practitioners. Unfortunately, the most popular spatial regression model, although intuitive as a posited data-generating mechanism, is problematic as a data-analytic tool. In this article I will describe a little-used alternative spatial regression model that has an equally satisfying motivation but avoids the challenges faced by the more popular model.

## Spatial regression models: two roads diverge

### A linear spatial regression model for Gaussian outcomes

Both of the spatial regression models treated herein have their genesis in the spatial linear mixed-effects regression model (SLMM), which can be specified as follows. We have

1 $$\begin{aligned} \varvec{Y}&= \mathbf {X}\varvec{\beta }+\varvec{\gamma }+\varvec{\varepsilon }, \end{aligned}$$

where $\varvec{Y}=(Y_1,\dots ,Y_n)'$ is the response; $\mathbf {X}_{n\times p}$ is the design matrix, the *p* columns of which are spatially structured covariates; $\varvec{\beta }=(\beta _1,\dots ,\beta _p)'$ are regression coefficients; $\varvec{\gamma }=(\gamma _1,\dots ,\gamma _n)'$ are spatially dependent random effects, the purpose of which is to accommodate spatial structure in the response that cannot be explained by $\mathbf {X}$; and $\varvec{\varepsilon }=(\varepsilon _1,\dots ,\varepsilon _n)'$ are iid Gaussian errors, which are independent of $\varvec{\gamma }$. Note that each element of $\varvec{Y}$, of $\varvec{x}_j\;(j=1,\dots ,p)$, of $\varvec{\gamma }$, and of $\varvec{\varepsilon }$ is spatially referenced, i.e., $Y_i=Y(\varvec{s}_i)$, $x_{ji}=x_j(\varvec{s}_i)$, $\gamma _i=\gamma (\varvec{s}_i)$, and $\varepsilon _i=\varepsilon (\varvec{s}_i)$, where $\varvec{s}_i$ is the *i*th spatial location at which the response and covariates were observed. The locations $\{\varvec{s}_i\}$ may be points in a continuous domain (e.g., latitude and longitude); or the locations may represent spatial aggregates (e.g., Census tracts, states, pixels), which are referred to as areal units. To increase readability I will generally omit the locations $\{\varvec{s}_i\}$.

Two characteristics make (1) a spatial regression model. First, the explanatory variables in $\mathbf {X}$ exhibit spatially patterned variation, as I mentioned above. Indeed, the chief aim of a spatial regression analysis is to explain spatially patterned variation in the response $\varvec{Y}$ as having arisen due to an association between $\varvec{Y}$ and one or more columns of $\mathbf {X}$. Second, the spatial random effects $\varvec{\gamma }$ accommodate/induce spatial variation in the response beyond that which can be attributed to $\mathbf {X}$. Indeed, one might say $\varvec{\gamma }$ is a stand-in for covariates that are missing from $\mathbf {X}$.

It is the supposed random nature of $\varvec{\gamma }$ that makes model (1) a mixed-effects model: $\varvec{\beta }$ are fixed effects and $\varvec{\gamma }$ are random effects. Typically, $\varvec{\gamma }$ is assumed to follow a multinormal distribution having mean $\varvec{0}$ and spatial covariance matrix ${\varvec{\Sigma }}_{n\times n}$. To say that ${\varvec{\Sigma }}$ is a *spatial* covariance matrix is to say that the $ii'$th element ($i\ne i'$) of ${\varvec{\Sigma }}$ accounts for the spatial relationship between locations $\varvec{s}_i$ and $\varvec{s}_{i'}$. For example, when the spatial domain of interest is continuous, it is common to let ${\varvec{\Sigma }}_{ii'}=\text {cov}(\gamma _i,\gamma _{i'})=K(\varvec{s}_i,\varvec{s}_{i'}\mid \varvec{\psi })$, where *K* is a spatial kernel function having parameters $\varvec{\psi }$. For some applications, an appealing choice of *K* is the powered exponential kernel, which is given by

$$\begin{aligned} K(\varvec{s}_i,\varvec{s}_{i'}\mid \nu ,\phi )= \exp \left\{ -\left( \frac{\vert \varvec{s}_i-\varvec{s}_{i'}\vert }{\phi }\right) ^\nu \right\} , \end{aligned}$$

where $\vert \varvec{s}_i-\varvec{s}_{i'}\vert$ is the distance between locations $\varvec{s}_i$ and $\varvec{s}_{i'}$, $\nu \in (0,2]$ is a smoothness parameter, and $\phi >0$ is a range parameter.

Note that models of the sort just described are usually referred to as Gaussian process (GP) models. GP methods are immensely popular not only in spatial statistics but more generally. The above described spatial model is referred to as a point-level model since the spatial locations are points in a continuous domain. Point-level models are often (less precisely) termed geostatistical models.

**Figure 2:**
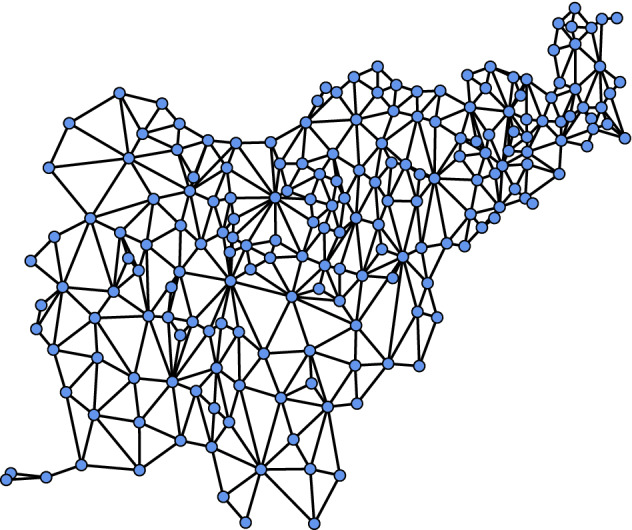
The adjacency structure $G=(V,E)$ for the municipalities of Slovenia. Figure created using R version 4.1.2 (https://www.r-project.org).

Should the spatial domain comprise aggregates (as for the Slovenia stomach cancer data presented above), ${\varvec{\Sigma }}$ is typically constructed from the undirected graph $G=(V,E)$ that represents the adjacency structure among the areal units (for Slovenia, municipalities; see Fig. 2). Here each areal unit corresponds to exactly one vertex in $V=\{1,2,\dots ,n\}$, and edge $(i,i')\in E$ if and only if areal units *i* and $i'$ are adjacent. A popular form of ${\varvec{\Sigma }}$ in this case is the proper conditional autoregressive (CAR) model^2^, for which

$$\begin{aligned} {\varvec{\Sigma }}=[\tau (\mathbf {D}-\rho \mathbf {A})]^{-1}, \end{aligned}$$

where $\tau >0$ is a smoothing parameter, $\mathbf {D}_{n\times n}$ is diagonal with the degrees of the vertices of *G* as its diagonal elements, $\rho \in [0,1)$ is a range parameter, and $\mathbf {A}_{n\times n}$ is the adjacency matrix for *G*, i.e., $\mathbf {A}_{ii'}=1\{(i,i')\in E\}$.

Note that the proper CAR and similar models are Gaussian Markov random field (GMRF) models, which is to say that, conditional on its neighbors, each outcome is independent of the remaining outcomes (spatial Markov property). The dependence structure for a GMRF model is specified in terms of a precision matrix rather than a covariance matrix. It is the form of the precision matrix that implies the conditional independency structure. Moreover, the precision matrix is sparse, and so fast sparse-matrix routines can often be used to speed computation.

The curious reader may desire more information on the methods I just touched on. An excellent book-length presentation of GP methods can be found in Williams and Rasmussen^3^. A seminal book in the field of spatial models, which also discusses GP methods but is more wide ranging, is Banerjee et al.^4^. See Rue and Held^5^ for a seminal book-length treatment of GMRF models. For a concise treatment of Gaussian random field models for spatial data, see Haran^6^. For a survey of Markov random field models and their applications, see Kindermann and Snell^7^. The seminal paper on geostatistical models is Diggle et al.^8^. And the pathbreaking paper on spatial Markov random field models is Besag^9^. Of course many other excellent sources exist.

In any case, there are many possibilities for ${\varvec{\Sigma }}$, and each of the many spatial linear mixed-effects regression models is distinguished by the manner in which said model constructs ${\varvec{\Sigma }}$ from $\varvec{s}_1,\dots ,\varvec{s}_n$. For each model ${\varvec{\Sigma }}$ is structured so that $\gamma _i$ and $\gamma _{i'}$—and hence $Y_i$ and $Y_{i'}$—exhibit stronger positive correlation the closer $\varvec{s}_i$ and $\varvec{s}_{i'}$ are to one another, and $\text {cor}(\gamma _i,\gamma _{i'})$ decays towards 0 as the distance between $\varvec{s}_i$ and $\varvec{s}_{i'}$ increases.

Finally, properties of the multinormal distribution imply that the response, too, is multinormal:

2 $$\begin{aligned} \varvec{Y}\sim \textsc {Normal}(\mathbf {X}\varvec{\beta },{\varvec{\Sigma }}+\sigma ^2\mathbf {I}), \end{aligned}$$

where $\sigma ^2\in \mathbb {R}^+$ is the common variance of the $\varepsilon _i$ and $\mathbf {I}$ denotes the $n\times n$ identity matrix. Thus the SLMM can be viewed as a linear regression model having spatially correlated multinormal errors as well as the iid Gaussian errors of the ordinary linear model, the latter of which represent extra-spatial dispersion, i.e., variation that cannot be attributed to the spatial process.

### Extending the linear spatial regression model

Should we desire to accommodate non-Gaussian response variables, the two formulations of the SLMM, (1) and (2), suggest two different approaches. The first form of the SLMM suggests that we model a non-Gaussian response as

3 $$\begin{aligned} \mathbb {E}(\varvec{Y}\mid \mathbf {X},\varvec{\gamma })&=g^{-1}(\mathbf {X}\varvec{\beta }+\varvec{\gamma }), \end{aligned}$$

where *g* is a suitable link function and the linear predictor $\mathbf {X}\beta +\varvec{\gamma }$ is the same as above. We pair (3) with an appropriate distribution for the response. The resulting model is called the spatial generalized linear mixed model (SGLMM) since it can be viewed as a generalized linear model^10,11^ that induces spatial dependence in the response variable by augmenting the ordinary linear predictor $\mathbf {X}\varvec{\beta }$ with spatially dependent random effects $\varvec{\gamma }$. (Conditional on $\varvec{\gamma }$, the outcomes are assumed to be independent).

The SGLMM is immensely popular—so popular, in fact, that alternatives receive little attention, despite the fact that the SGLMM poses a number of formidable challenges (to be described shortly).

The second form of the SLMM suggests an alternative to the SGLMM, namely, the spatial direct Gaussian copula regression model (SGCRM). The SGCRM can be specified as

4 $$\begin{aligned} \varvec{Z}&=(Z_1,\dots ,Z_n)' \sim \textsc {Normal}(\varvec{0},{\varvec{\Omega }})\nonumber \\ U_i&=\Phi (Z_i) \sim \textsc {Uniform}(0,1)\;\;\;(i=1,\dots ,n)\nonumber \\ Y_i&=F_i^{-1}(U_i) \sim F_i(\cdot \mid \varvec{x}_i), \end{aligned}$$

where ${\varvec{\Omega }}$ is a spatial correlation matrix, $F_i$ and $\varvec{x}_i=(x_{i1},\dots ,x_{ip})'$ are the cdf and covariates, respectively, for the response variable $Y_i$, and $F_i^{-1}$ is the quantile function for $F_i$. Note that the $U_i$ are marginally standard uniform owing to the probability integral transform, and $Y_i$ follows distribution $F_i$ owing to the inverse probability integral transform^12^.

In this scheme we have

$$\begin{aligned} \mathbb {E}(\varvec{Y}\mid \mathbf {X})=g^{-1}(\mathbf {X}\varvec{\beta }) \end{aligned}$$

for an appropriate choice of $F_1,\dots ,F_n$. Thus we see that the SGCRM induces “extra” spatial dependence “from below,” if you will, as opposed to augmenting the ordinary linear predictor. This extra spatial structure is produced by a Gaussian spatial copula, of which $\varvec{U}=(U_1,\dots ,U_n)'$ is a realization: the $U_i$ are marginally standard uniform and jointly carry the dependence structure encoded in ${\varvec{\Omega }}$. (For book-length treatments of copula methods see Nelsen^13^ and/or Joe^14^. And excellent papers on the subject are Kolev and Paiva^15^ and Xue-Kun Song^16^).

Note that the SGLMM is equivalent to the SLMM if *g* is the identity function and the response distributions are Gaussian and have covariance matrix $\sigma ^2\mathbf {I}$. Likewise, the SGCRM is equivalent to the SLMM if ${\varvec{\Omega }}$ is the correlation matrix corresponding to ${\varvec{\Sigma }}$ and the response distributions are Gaussian with means $\varvec{x}_i'\varvec{\beta }$ and variances ${\varvec{\Sigma }}_{ii}+\sigma ^2$. Thus the SLMM and the SGCRM are the same model in this case.

When the response is non-Gaussian, the SGLMM and the SGCRM are usually not equivalent^17^, and so for a non-Gaussian SGLMM we must consider the joint distribution $f(\varvec{Y},\varvec{\gamma })=f(\varvec{Y}\mid \varvec{\gamma })f(\varvec{\gamma })$, while the SGCRM is a marginal model irrespective of the response distribution. This implies that $\varvec{\beta }$ has a conditional interpretation for non-Gaussian SGLMMs, i.e., $\varvec{\beta }$ has its usual interpretation only conditional on the spatial random effects $\varvec{\gamma }$ since the random vector $\varvec{\gamma }$ is essentially an additional, but unobserved, covariate. By contrast, the SGCRM and the ordinary generalized linear model share the same intuitive marginal interpretation of $\varvec{\beta }$ because both models employ the “bare” linear predictor $\mathbf {X}\varvec{\beta }$ instead of $\mathbf {X}\varvec{\beta }+\varvec{\gamma }$.

And the problem of interpretation does not end there, for the SGLMM exhibits spatial confounding, a type of perfect collinearity between $\varvec{\gamma }$ and $\mathbf {X}$ that can lead to a rather different value for $\hat{\varvec{\beta }}$ and inflate $\hat{\varvec{\beta }}$’s standard errors so dramatically as to conceal important associations between $\varvec{Y}$ and $\mathbf {X}$^18,19^. To see why the SGLMM is spatially confounded, first let $\mathbf {P}$ be the orthogonal projection onto $C(\mathbf {X})$ (the column space of $\mathbf {X}$), so that $\mathbf {I}-\mathbf {P}$ is the orthogonal projection onto $C(\mathbf {X})^\perp$. Now eigendecompose $\mathbf {P}$ and $\mathbf {I}-\mathbf {P}$ to obtain orthogonal bases ($\mathbf {K}_{n\times p}$ and $\mathbf {L}_{n\times (n-p)}$, say) for $C(\mathbf {X})$ and $C(\mathbf {X})^\perp$. Then the augmented linear predictor can be rewritten as

$$\begin{aligned} \mathbf {X}\varvec{\beta }+\varvec{\gamma }=\mathbf {X}\varvec{\beta }+\mathbf {K}\varvec{\delta }+\mathbf {L}\varvec{\eta }, \end{aligned}$$

where $\varvec{\delta }_{p\times 1}$ and $\varvec{\eta }_{(n-p)\times 1}$ are random coefficients. This form shows that $\mathbf {K}$ is the source of the confounding, for $\mathbf {K}$ and $\mathbf {X}$ have the same column space.

So troubling is spatial confounding that a considerable literature devoted to the problem now exists. Yet no proposed remedy appears to be satisfactory, and it may be safe to conclude that no proper remedy will ever be found within the standard mixed-effects paradigm^20^. It is fortunate that the SGCRM does not suffer from spatial confounding (because the SGCRM’s linear predictor does not contain spatial random effects).

Clayton et al.^18^ discovered spatial confounding. Reich et al.^19^ and Paciorek^21^ then sparked renewed (and sustained) interest in spatial confounding, and offered means of alleviating or remedying the problem. Since then many articles have appeared on the subject. Many of those articles propose some remedy or other *within the mixed-effects paradigm*. Instead, I recommend avoiding the problem altogether by setting aside the mixed-effects paradigm (for regression).

Some spatial modelers might contend that we simply must work within the mixed-effects paradigm if we aim to do both spatial regression and spatial smoothing. But the literature on spatial confounding suggests that regression and smoothing are cross purposes. Perhaps we should consider spatial regression and spatial smoothing to be distinct tasks, and treat them as such. In the remainder of this article I will put forth the SGCRM as a compelling solution to the spatial regression problem. I will address spatial smoothing in future work.

Finally, I would be remiss if I did not mention an additional, and important, potential pathology of the SGLMM, namely, that the conclusions implied by an SGLMM fit can be implausible not only for the regression but also for the dependence model. In a later section I will demonstrate by applying an SGLMM to the Slovenia data. For those data, results for both the regression part of the model and the dependence part of the model are implausible.

## Approaches to inference for the SGCRM

If one aims to estimate simultaneously the marginal parameters and the copula parameters, doing inference for the SGCRM does not permit a unified approach. For continuous outcomes the SGCRM likelihood is meta-Gaussian, and so likelihood-based inference (i.e., maximum likelihood or Bayesian inference) is straightforward—but potentially burdensome computationally owing to repeated computation of $\vert {\varvec{\Omega }}\vert$ and ${\varvec{\Omega }}^{-1}$ along with simultaneous estimation of the marginal parameters. For discrete outcomes the SGCRM likelihood comprises on the order of $2^n$ terms and is thus intractable for realistic sample sizes. Consequently, a number of tractable alternative objective functions have been proposed for discrete data^22–28^. Although these alternatives are compelling and well-studied, I recommend a simple, flexible, unified (i.e., suitable for both continuous and discrete outcomes) two-stage approach to inference, as follows.

Recall that the standardized residuals for an ordinary GLM fit are given by

$$\begin{aligned} r_i=\frac{e_i}{\sqrt{1-\hat{h}_i}} \end{aligned}$$

or

$$\begin{aligned} r_i=\frac{d_i}{\sqrt{1-\hat{h}_i}}, \end{aligned}$$

where $e_i$ is the Pearson residual for the *i*th outcome $Y_i$, $d_i$ is the corresponding deviance residual, and $\hat{h}_i$ is the estimated leverage of $Y_i$. Since these residuals are asymptotically standard normal for most forms of the GLM^11,29^, it is quite natural in a multivariate setting to regard $\varvec{r}=(r_1,\dots ,r_n)'$ as a realization of $\varvec{Z}$ from (4). (In fact, it is advantageous to regard $\varvec{r}$ as a realization of $\varvec{Z}$ even when the residuals appear to depart markedly from standard normality—more on this later.) This suggests that we can fit an SGCRM by first fitting an ordinary GLM having linear predictor $\mathbf {X}\varvec{\beta }$, and then using standardized residuals to estimate the parameters of ${\varvec{\Omega }}$. Specifically, we can estimate ${\varvec{\Omega }}$ by optimizing

$$\begin{aligned} \ell ({\varvec{\Omega }}\mid \varvec{r})=-\frac{1}{2}\log \vert {\varvec{\Omega }}\vert -\frac{1}{2}\varvec{r}'{\varvec{\Omega }}^{-1}\varvec{r}. \end{aligned}$$

Finally, armed with $\hat{\varvec{\beta }}$ and $\hat{{\varvec{\Omega }}}$, we can do parametric bootstrap^30,31^ inference for $\varvec{\beta }$, as follows.

1. Fit an ordinary GLM to $\varvec{Y}$ to estimate the marginal parameters $\varvec{\beta }$ (and perhaps additional, nuisance, parameters).
2. Use the standardized residuals from Step 1 to estimate the parameters of ${\varvec{\Omega }}$.
3. Produce a bootstrap sample $\hat{\varvec{\beta }}^*_1,\dots ,\hat{\varvec{\beta }}^*_{n_b}$ for $\hat{\varvec{\beta }}$, where $n_b$ is the bootstrap sample size, as follows. For $k\in \{1,\dots ,n_b\}$,
  - simulate $$\varvec{Z}^*_k\sim \textsc {Normal}(\varvec{0},\hat{{\varvec{\Omega }}})$$;
  - compute $\varvec{U}^*_k$ by applying the probability integral transform—as in (4) above—to $\varvec{Z}^*_k$;
  - produce $\varvec{Y}^*_k$ by applying the inverse probability integral transform—as in (4) above—to $\varvec{U}^*_k$, where the *i*th quantile function has parameters $\hat{\varvec{\beta }}$ (and perhaps estimates of nuisance marginal parameters);
  - obtain $\hat{\varvec{\beta }}^*_k$ by fitting the above mentioned ordinary regression model to $\varvec{Y}^*_k$.

The resulting bootstrap sample reflects the more dispersed—that is, more variable due to reduced effective sample size—sampling distribution of $\hat{\varvec{\beta }}$ under dependence and so can be used to do much improved inference for $\varvec{\beta }$.

## Application of the SGCRM to simulated data

For any topic as broad as spatial regression, many satisfactory simulation study designs exist. I feel that the study design I used for this article more than accomplishes my goal, namely, to exercise broadly my proposed methodology by applying the methodology in a variety of realistic scenarios. To that end, I considered both areal and continuous-domain data; the most common response types; various realistic sample sizes; common forms of the spatial Gaussian copula; both strong and weaker spatial dependence; and intuitive spatially patterned covariates (which permit easy visualization). I considered six scenarios (see Table 1).

The first three scenarios were for areal (i.e., spatially aggregated) versions of the SGCRM. For Scenarios 1 and 2 I used a proper CAR copula and Poisson marginal distributions. For both scenarios the underlying graph was the $30\times 30$ square lattice (sample size $n=900$). Recall that the proper CAR model has precision matrix proportional to $\mathbf {D}-\rho \mathbf {A}$, where parameter $\rho \in [0,1)$ is a range parameter. I used two values for $\rho$, namely, 0.99 in Scenario 1 and 0.8 in Scenario 2. These values represent strong spatial dependence and somewhat weaker (but still consequential) dependence, respectively.

**Figure 3:**
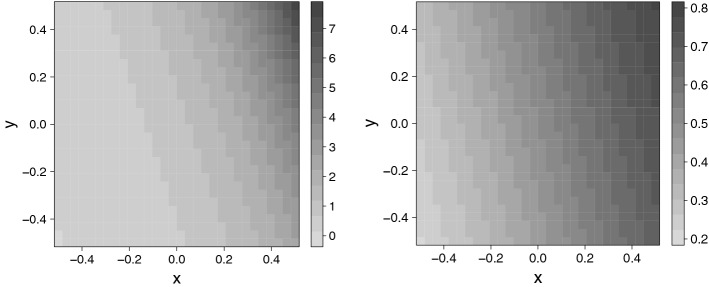
Left panel: the mean structure for the Poisson (Scenarios 1 and 2) and negative binomial (Scenarios 5 and 6) simulation studies. Right panel: the mean structure for the binomial (Scenario 3) and ungrouped binary (Scenario 4) simulation studies. Figure created using R version 4.1.2 (https://www.r-project.org).

I assigned to the lattice points locations in the unit square centered at the origin, so that both the *x* and *y* coordinates range from $-0.5$ to 0.5. I used these coordinates as the spatial explanatory variables by using Poisson rates $\exp (\beta _1x_i+\beta _2y_i)\;(i=1,\dots ,900)$, where $\beta _1$ is an east–west effect and $\beta _2$ is a north–south effect. For both scenarios I used $\beta _1=3$ (strong effect) and $\beta _2=1$ (weaker effect). The resulting mean structure is shown in the left panel of Fig. 3.

Scenario 3 increased the sample size to $n=$ 1,600 by using the $40\times 40$ lattice. And I used the Leroux GMRF specification^32^ for the copula in Scenario 3. The Leroux precision matrix is a mixture of the $n\times n$ identity matrix and the intrinsic CAR precision matrix, which is proportional to $\mathbf {D}-\mathbf {A}$. (The intrinsic CAR is obtained by setting $\rho$ equal to 1 in the proper CAR model. The intrinsic CAR is widely considered to be an appealing prior distribution in Bayesian spatial modeling, but we cannot use the intrinsic CAR in the SGCRM because $\mathbf {D}-\mathbf {A}$ is singular.) Specifically, the Leroux precision matrix is proportional to

$$\begin{aligned} (1-\lambda )\mathbf {I}+\lambda (\mathbf {D}-\mathbf {A}), \end{aligned}$$

where $\mathbf {I}$ is the identity matrix and $\lambda \in [0,1)$ is a spatial dependence parameter. This specification yields the independence case if $\lambda =0$, and approaches the intrinsic autoregression as $\lambda \rightarrow 1$. I used $\lambda =0.95$, which implies strong dependence. (See Waller and Carlin^33^, Lee^34^, and/or LeSage and Pace^35^ for more information regarding the proper CAR, Leroux, and similar models).

As for response distribution and mean structure, I used binomial marginals for Scenario 3, where the subsample size was $N=20$ and the “success” probabilities were

$$\begin{aligned} \pi _i=\frac{\exp (\beta _1x_i+\beta _2y_i)}{1 + \exp (\beta _1x_i+\beta _2y_i)}\;\;(i=1,\dots ,\text {1,600}). \end{aligned}$$

I let $\beta _1=2$ and $\beta _2=0.5$, which produced $\pi _i$ over the range 0.2 to 0.8, approximately. This mean structure is shown in the right panel of Fig. 3.

The final three scenarios employed Gaussian process specifications for their copulas. For Scenarios 5 and 6 I used a two-parameter Matérn spatial correlation function^36,37^. This correlation function is given by

$$\begin{aligned} K(\varvec{s}_i,\varvec{s}_{i'}\mid \phi ,\nu )=\frac{2^{1-\nu }}{\Gamma (\nu )}\left( \frac{|\varvec{s}_i-\varvec{s}_{i'}|}{\phi }\right) ^\nu K_\nu \left( \frac{|\varvec{s}_i-\varvec{s}_{i'}|}{\phi }\right) , \end{aligned}$$

where $\phi >0$ is a range parameter, $\nu >0$ is a smoothness parameter, $\Gamma$ denotes the gamma function, and $K_\nu$ denotes the modified Bessel function of the third kind of order $\nu$. This kernel is immensely popular not only in spatial statistics but also in machine learning, computer experiments^38^, and elsewhere. I set $\nu$ equal to 1 for both of Scenarios 5 and 6, and I let $\phi =0.1$ for Scenario 5 and $\phi =0.03$ for Scenario 6. These parameter settings correspond to strong dependence and weaker (yet consequential) dependence, respectively.

For simulation Scenarios 5 and 6 I once again used the mean structure shown in the left panel of Fig. 3, but I used a less dense lattice ($20\times 20$), and I chose negative binomial marginal distributions. The negative binomial is an important model for spatial counts because the negative binomial distribution accommodates overdispersion, i.e., variance larger than the mean, which is commonly exhibited by count data in many contexts but cannot be handled by the equi-dispersed Poisson distribution. I used the parameterization of the negative binomial distribution that provides variance function $v(\mu )=\mu +\mu ^2/\theta$, where $\mu$ is the mean and $\theta >0$ is the dispersion parameter. For $\theta$ close to 0 the variance is much larger than the mean, and as $\theta \rightarrow \infty$ the negative binomial distribution converges to the Poisson distribution. I used $\theta =3$ for both scenarios. This value of $\theta$ produces moderate overdispersion.

Finally, for simulation Scenario 4 I employed a special case of the Matérn correlation function, namely, the exponential correlation function:

$$\begin{aligned} K(\varvec{s}_i,\varvec{s}_{i'}\mid \phi )=\exp \left( -3\,\frac{|\varvec{s}_i-\varvec{s}_{i'}|}{\phi }\right) . \end{aligned}$$

This kernel is suitable for ungrouped binary data since it fixes the smoothness $\nu$ (at 0.5), leaving only the range parameter $\phi$ to be estimated. Learning the smoothness is hopeless for binary data, and so it makes sense to assume a rough (merely continuous in the mean-squared sense) process. I used $\phi =0.3$, which implies strong dependence. Note that I included 3 in the exponential kernel because doing so lends an intuitive interpretation to the range parameter: $\phi$ is the so-called effective range of the process, i.e., the distance between locations $\varvec{s}_i$ and $\varvec{s}_{i'}$ at which $\text {cor}(Z_i,Z_{i'})$ has fallen to 0.05.

Because my simulation study design melds areal methodology and point-level methodology, it may seem as if my design employs the same spatial domain for both areal data and point-level data. This is not the case, however. For areal data, the underlying graph—in this case a square grid graph—is the spatial domain. For point-level data, the outcomes are observed on a square grid of locations covering the unit square centered at the origin, and so said square is the spatial domain. Because these two notions of spatial domain (discrete domain versus continuous domain) are rather different, areal copulas and point-level copulas are specified in rather different ways.

Note that I did not consider continuous response distributions in the simulation. Results for continuous outcomes are predictable because residuals are very well behaved for continuous-response GLMs. And so I chose to focus on discrete spatial data, which are common and pose more of a challenge for any spatial method.

Also note that I used a bootstrap sample size of 1,000 and a simulation sample size (i.e., number of simulated datasets) of 1,000 for all simulations described in this article.

**Table 1:** Scenarios for the simulation study.

Scenario	Response	Copula	Sample size	Copula parameter(s)	$\varvec{\beta }$
1	Poisson	Proper CAR	$30\times 30$	$\rho =0.99$ (strong)	$\beta _1 = 3, \beta _2=1$
2				$\rho =0.8$ (weaker)	
3	Binomial $(N=20)$	Leroux	$40\times 40$	$\lambda =0.95$ (strong)	$\beta _1 = 2, \beta _2=0.5$
4	Bernoulli	Exponential	$30\times 30$	$\phi =0.3$ (strong)	$\beta _1 = 2, \beta _2=0.5$
5	Negative binomial	Matérn	$20\times 20$	$\phi =0.1,\nu =1$ (strong)	$\beta _1 = 3, \beta _2=1$
6				$\phi =0.03,\nu =1$ (weaker)	

The regression results from the simulation study are given in Table 2. For Scenario 1 (Poisson marginals, proper CAR copula, strong dependence) and 95% intervals we see that the coverage rates for ordinary GLM intervals was very poor (27%, 32%) while the coverage rates for the spatial methodology were much better (84%, 88%).

It is unfortunate but not surprising that the spatial method did not provide the desired 95% coverage; this scenario is challenging due to the quite strong dependence ($\rho =0.99$). This can be remedied, however, by using 99% intervals instead (second row of Table 2): we see that 99% intervals gave 95% coverage for both regression coefficients. This could be seen as a weakness of the proposed methodology, but at least the deficiency can be remedied, and the use of similar remedies is commonplace in other statistical contexts. For example, when applying local polynomial methods it is good practice to intentionally undersmooth since the bandwidth chosen by cross-validation tends to be a bit too large. Using a slightly smaller bandwidth yields more accurate pointwise confidence bands.

For Scenario 2 (Poisson marginals, proper CAR copula, moderate dependence) the spatial methodology produced 95% intervals having the desired coverage rate. This shows just how challenging Scenario 1 was.

The results for Scenario 3 (binomial marginals, Leroux copula, strong dependence) were similar to the results for Scenario 1. The ordinary GLM intervals had very poor coverage rates while the spatial intervals performed much better. Once again the 95% spatial intervals did not have the desired coverage rate (91%, 90%), but 99% intervals offered better than 95% coverage (98%, 98%).

For Scenario 4 (ungrouped binary outcomes, exponential copula, strong dependence), which is certainly the most challenging scenario, the spatial methodology performed rather poorly. This is what I expected. I included this scenario in the study in the interest of completeness and also to show just how bleak are our prospects for revealing the data-generating mechanism behind dependent ungrouped binary data. I plan to make dependent ungrouped binary data the focus of a future study.

For the final two simulation scenarios, Scenarios 5 and 6, (negative binomial marginals, Matérn copula, strong and moderate dependence, respectively) we see that once again the spatial methodology performed much better than the ordinary GLM methodology. And once again 99% confidence intervals were required in the case of strong dependence (Scenario 5). For Scenario 6, 95% intervals performed well (97% coverage for $\beta _1$ and 95% coverage for $\beta _2$) owing to weaker, but still consequential, spatial dependence.

For all scenarios the findings for type II error rates (type II error defined as interval contains 0) were predictable. Specifically, type II error rates are higher for the spatial method than for the ordinary GLM. This is because my proposed method yields wider confidence intervals, reflecting the reduced effective sample size caused by positive spatial dependence.

Average running times were short—considerably shorter than the running times for competing Bayesian procedures or for frequentist approaches that estimate the marginal parameters and the copula parameters simultaneously. Note that the reported average running times were for the full approach as outlined above (estimation of marginal parameters, followed by estimation of copula parameters, followed by bootstrapping). I did not parallelize the bootstrap sampling, however. An embarrassingly parallelized^39^ bootstrap procedure would of course lead to even shorter running times.

**Table 2:** Regression results for the simulation scenarios described in Table 1.

Scenario	Interval (%)	Parameter	Ordinary coverage rate (%)	Spatial coverage rate (%)	Ordinary type II rate (%)	Spatial type II rate (%)	Running time (s)
1	95	$\beta _1$	27	84	0	0	11
		$\beta _2$	32	88	3	29	
	99	$\beta _1$	–	95	–	0	
		$\beta _2$	–	95	–	50	
2	95	$\beta _1$	70	94	0	0	10
		$\beta _2$	69	94	0	0	
3	95	$\beta _1$	29	91	0	0	48
		$\beta _2$	31	90	2	26	
	99	$\beta _1$	–	98	–	0	
		$\beta _2$	–	98	–	48	
4	95	$\beta _1$	31	59	6	13	16
		$\beta _2$	36	63	30	58	
5	95	$\beta _1$	31	89	0	7	101
		$\beta _2$	31	88	16	68	
	99	$\beta _1$	–	95	–	12	
		$\beta _2$	–	94	–	80	
6	95	$\beta _1$	72	97	0	0	90
		$\beta _2$	70	95	2	19	

Simulation results for the nuisance parameters—$\rho$, $\lambda$, $\phi$, $\nu$, and $\theta$—are shown in Table 3. For Scenarios 1 and 2, the proper CAR scenarios, we see that the copula range parameter $\rho$ can be recovered with little bias despite the fact that the outcomes for those scenarios were discrete (Poisson). For Scenario 2 the estimator $\hat{\rho }$ is approximately normally distributed because the value of the parameter, $\rho =0.8$, is sufficiently far from 1 and the sample size was on the large side for areal data. For Scenario 1 the estimator $\hat{\rho }$ is left-skewed because the value of the parameter, $\rho =0.99$, is rather close to 1. If desired, an approximately Gaussian estimator can be obtained as $\Phi ^{-1}(\hat{\rho })$, where $\Phi ^{-1}$ is the standard normal quantile function. This is useful for obtaining a symmetric confidence interval for $\Phi ^{-1}(\hat{\rho })$, the endpoints of which can be transformed back to the scale of $\rho$ to obtain an interval for $\rho$.

For the Leroux copula in Scenarion 3, where the response distribution was binomial, once again the dependence parameter $\lambda$ can be estimated with only a small bias. And $\hat{\lambda }$’s left-skewed distribution can be transformed to approximate Gaussianity by applying the standard normal quantile function, if necessary.

The first Matérn scenario, Scenario 4, yielded a poor result, as expected. For ungrouped binary data, neither marginal parameters nor dependence parameters can be recovered reliably. Specifically, in Scenario 4 the median estimate of the Matérn range parameter $\phi$ was biased downward by 63%. That is, the underlying spatial dependence appears to be much weaker than it is. This implies that the effective sample size is overestimated, which leads to very optimistic inference for the regression coefficients. In a future project I will further explore dependent ungrouped binary data.

For the negative binomial outcomes in simulation Scenario 5, the range parameter can be recovered with only a small positive bias. But $\hat{\nu }$ is substantially biased downward because information about spatial smoothness is lost to discretization. Also note that $\hat{\theta }$ exhibited a large upward bias for this scenario. This can be attributed to the dependence structure: strong positive dependence reduces the variance of the outcomes, working against the overdispersion induced by the marginal distributions.

In Scenario 6 smoothness parameter $\nu$ was once again substantially underestimated, on average. And $\hat{\phi }$ again exhibited positive bias, which was small in magnitude but large as a percentage of the true parameter value. Finally, because the dependence was considerably weaker for Scenario 6, dispersion estimator $\hat{\theta }$ was only slightly biased upward.

**Table 3:** Results for nuisance parameters for the simulation scenarios described in Table 1.

Scenario	Parameter	Median estimate	Remarks
1	$\rho =0.99$	0.976	Small negative bias; $\hat{\rho }$ has left-skewed distribution
2	$\rho =0.8$	0.763	Small negative bias; $\hat{\rho }$ is approximately normally distributed
3	$\lambda =0.95$	0.931	Small negative bias; $\hat{\lambda }$ has slightly left-skewed distribution
4	$\phi =0.3$	0.109	Large negative bias (63%); $\hat{\phi }$ has slightly right-skewed distribution
5	$\phi =0.1$	0.114	Small bias; $\hat{\phi }$ has strongly right-skewed distribution
	$\nu =1$	0.654	Substantial negative bias; $\hat{\nu }$ has right-skewed distribution
	$\theta =3$	5.533	Positive bias; distribution of $\hat{\theta }$ has a heavy right tail; many values larger than 10; some extreme values
6	$\phi =0.03$	0.047	Positive bias; $\hat{\phi }$ has a right-skewed distribution; a few extreme values
	$\nu =1$	0.509	Large negative bias; $\hat{\nu }$ has a strongly right-skewed distribution (range 0–6)
	$\theta =3$	3.286	Small positive bias; $\hat{\theta }$ has a right-skewed distribution

## Model assessment and choice

Although standardized residuals are quite useful for estimating copula parameters, standardized residuals are often not terribly useful for assessing model appropriateness when the response variable is discrete. Specifically, standardized residuals for discrete outcomes may be far from Gaussian and exhibit banding. Consequently, I recommend that standardized residuals be used only for estimating copula parameters. For assessing model fit I strongly recommend the use of randomized quantile residuals (RQR)^40^ since Feng et al.^41^ recently showed that RQRs are powerful for detecting many forms of misspecification (e.g., nonlinear effects of covariates, overdispersion, zero inflation) for discrete regression models. Note, however, that RQRs should not be used in place of standardized residuals when estimating copula parameters, for the production of RQRs somewhat obscures the dependence structure, leading to poor estimation of ${\varvec{\Omega }}$. I discovered this fact through simulation.

Akaike’s information criterion (AIC)^42^ for ordinary GLMs—which is given by

$$\begin{aligned} \text {AIC}_\textsc {glm}=2p-2\hat{\ell }_\textsc {glm}, \end{aligned}$$

where *p* is the number of marginal parameters and $\hat{\ell }_\textsc {glm}$ denotes the maximum value of the ordinary GLM log-likelihood—is very useful for choosing the right response family, despite the fact that ordinary AIC neglects dependence. For example, in a large simulation study where the true response distributions were negative binomial and dependence was strong (Scenario 1 from Table 1, but with negative binomial outcomes according to Scenarios 5 and 6), AIC$_\textsc {glm}$ selected the true model over a Poisson model for every simulated dataset. In fact, the minimum difference in AIC values for said study was 23, and the average difference was 104. These are huge differences and so left little doubt that the negative binomial model was superior to the Poisson model.

Selecting the best copula model from a collection of candidates is more challenging. An approach that is analogous to using AIC$_\textsc {glm}$ to select a response model, one can use a version of AIC (or some other suitable information criterion^43^) to select a copula model. Specifically, one can compute

$$\begin{aligned} \text {AIC}_\textsc {cop}=2q-2\hat{\ell }_\textsc {cop}=2q+\log \vert \hat{{\varvec{\Omega }}}\vert +\varvec{r}'\hat{{\varvec{\Omega }}}^{-1}\varvec{r}, \end{aligned}$$

where *q* is the number of copula parameters, $\hat{{\varvec{\Omega }}}$ is the estimated copula correlation matrix, and $\varvec{r}$ are the standardized residuals for the first-stage fit selected using AIC$_\textsc {glm}$.

In the simulation study mentioned above, I used the CAR copula as the true copula and used AIC$_\textsc {cop}$ to choose between the CAR copula and the Leroux copula. Although a paired *t* test with unequal variances found a statistically significant difference ($\alpha =0.05$) between AIC$_\textsc {cop}$ for the true copula and AIC$_\textsc {cop}$ for the Leroux copula, the difference in means was very small and AIC$_\textsc {cop}$ selected the true copula for only 59% of the simulated datasets.

To determine whether these poor results should be taken as an indictment of AIC$_\textsc {cop}$ or of the CAR and Leroux copulas, I carried out a follow-up simulation study (for Scenarios 1 and 2) in which I computed AIC$_\textsc {cop}$ using simulated realizations of $\varvec{Z}$ (according to (4)) instead of using standardized residuals from GLM fits to simulated $\varvec{Y}$. The follow-up study yielded comparable results, and so we must conclude not that AIC$_\textsc {cop}$ loses information to discretization for areal models but that the CAR and Leroux models are practically indistinguishable. To my knowledge, this is a new finding.

Selecting a Gaussian process copula is more complicated. In a simulation study that employed negative binomial marginals and a Matérn copula with smoothness $\nu =1$ and range $\phi =0.1$, AIC$_\textsc {cop}$ chose the true copula over a powered exponential copula for only 30% of the simulated datasets. The percentage rose to 65% (considerably better but still far from outstanding performance) when I used simulated $\varvec{Z}$ instead of simulated outcomes. This shows that we do lose to discretization a substantial amount of information about the smoothness of a Matérn copula. This is not surprising and agrees with the results for Scenarios 5 and 6 from Table 3. I obtained comparable results when I simulated data according to a powered exponential copula and compared AIC$_\textsc {cop}$ for the true copula and a Matérn copula, and so it seems that the methodology described in this paper is fairly insensitive to the choice of GP copula, at least for rougher spatial processes.

A thoughtful reviewer suggested that I should also investigate the effect of copula misspecification on regression inference. I did so by simulating data according to Scenarios 1 and 2 (CAR copula), and fitting the Leroux copula. Both coverage rates and type II rates were unaffected. Specifically, for $\rho =0.8$ the coverage rates were 95% and 93%, and the type II rates were 0%. For $\rho =0.99$ the coverage rates were 86% and 88%, and the type II rates were 0% and 31%. I obtained very similar results for data simulated according to Scenarios 5 and 6. That is, regression inference was little affected by GP copula misspecification.

For smoother GP processes the Matérn model is preferred to the powered exponential model because the former is more flexible. (Note that the powered exponential and the Matérn coincide when the powered exponential has smoothness 1 and the Matérn has smoothness 0.5 (this is the exponential correlation function), and when the powered exponential has smoothness 2 and the Matérn has smoothness $\infty$ (this is the Gaussian or squared exponential correlation function).) Specifically, the process for the powered exponential kernel is not mean-squared differentiable except for smoothness $\nu =2$ (when the process is infinitely mean-squared differentiable). A Matérn process, on the other hand, is *m* times mean-squared differentiable iff $\nu >m$. In any case, Williams and Rasmussen^3^ pointed out that it is very difficult or even infeasible to distinguish between Matérn smoothness values larger than, say, 7/2. It is even difficult to distinguish between finite values of $\nu$ and $\nu \rightarrow \infty$ for the Matérn! And of course these difficulties are exacerbated when the response is discrete.

## Additional computing concerns

Naive computation of $|{\varvec{\Omega }}|$ and ${\varvec{\Omega }}^{-1}$ is burdensome and does not scale well. Thus it may be advantageous, or even necessary, to consider approaches for optimizing $\ell ({\varvec{\Omega }}\mid \varvec{r})=-\frac{1}{2}\log |{\varvec{\Omega }}|-\frac{1}{2}\varvec{r}'{\varvec{\Omega }}^{-1}\varvec{r}$ more efficiently. I will discuss efficient computing for areal models first, and then turn to continuous-domain variants of the SGCRM.

### Efficient computing for areal copulas

Recall that Gaussian copulas for areal data are typically Gaussian Markov random fields, which are parameterized in terms of their precision matrices. I considered two such models above, namely, the proper CAR model and the Leroux model. These models have precision matrices that are proportional to $\mathbf {Q}=\mathbf {D}-\rho \mathbf {A}$ and $\mathbf {Q}=(1-\lambda )\mathbf {I}+\lambda (\mathbf {D}-\mathbf {A})$, respectively. Efficient computing for these and similar models can be done as follows. I will use the proper CAR model as an example.

Note that $\mathbf {Q}$ is not an inverse correlation matrix because the variances ${\mathrm {vecdiag}}(\mathbf {Q}^{-1})$ are not equal to 1. But of course we can rescale $\mathbf {Q}$ so its inverse is a correlation matrix, i.e., we can construct a Gaussian copula using ${\varvec{\Omega }}^{-1}=\mathbf {V}^{1/2}\mathbf {Q}\mathbf {V}^{1/2}$, where $\mathbf {V}={\mathrm {diag}}({\mathrm {vecdiag}}(\mathbf {Q}^{-1}))$. This leads to objective function

$$\begin{aligned} \ell ({\varvec{\Omega }}\mid \varvec{r})&= \frac{1}{2}\log |{\varvec{\Omega }}^{-1}|-\frac{1}{2}\varvec{r}'{\varvec{\Omega }}^{-1}\varvec{r}\\&=\frac{1}{2}\log |\mathbf {V}^{1/2}\mathbf {Q}\mathbf {V}^{1/2}|-\frac{1}{2}\varvec{r}'\mathbf {V}^{1/2}\mathbf {Q}\mathbf {V}^{1/2}\varvec{r}\\&=\frac{1}{2}\log \vert \mathbf {Q}\vert +\frac{1}{2}\log \vert \mathbf {V}\vert -\frac{1}{2}\varvec{w}^\prime \mathbf {Q}\varvec{w}\\&=\frac{1}{2}\log \vert \mathbf {Q}\vert +\frac{1}{2}\sum _{i=1}^n\log {\mathrm {vecdiag}}(\mathbf {Q}^{-1})_i-\frac{1}{2}\varvec{w}^\prime \mathbf {Q}\varvec{w}\end{aligned}$$

where $\varvec{w}=\mathbf {V}^{1/2}\varvec{r}=\sqrt{{\mathrm {vecdiag}}(\mathbf {Q}^{-1})}\circ \varvec{r}$, with $\circ$ denoting the Hadamard product.

Now, numerical methods for sparse matrices can be used to compute $\vert \mathbf {Q}\vert$ quickly. Let $\mathbf {C}$ be the lower Cholesky triangle of $\mathbf {Q}$, so that $\mathbf {Q}=\mathbf {C}\mathbf {C}^\prime$. Then $\vert \mathbf {Q}\vert =\vert \mathbf {C}\vert \vert \mathbf {C}^\prime \vert =\vert \mathbf {C}\vert ^2$, which implies that

5 $$\begin{aligned} \frac{1}{2}\log \vert \mathbf {Q}\vert =\sum _{i=1}^n\log \mathbf {C}_{ii}. \end{aligned}$$

The righthand side of (5) can be computed efficiently because $\mathbf {C}$ can be computed efficiently after $\mathbf {Q}$ has been reordered to reduce its bandwidth. Also note that $\mathbf {C}$ needs to be computed just once—the structure of $\mathbf {C}$ depends on the sparsity structure of $\mathbf {Q}$ and not on $\rho$, and so $\mathbf {C}$ can simply be updated to reflect a change in $\rho$^44^.

It remains to handle the proper CAR variances efficiently. Since computing ${\mathrm {vecdiag}}(\mathbf {Q}^{-1})$ obviously requires inversion of $\mathbf {Q}$, it would seem that using the proper CAR copula must leave us unable to fully exploit the sparsity of $\mathbf {Q}$. This is not the case, however, because the variances ${\mathrm {vecdiag}}(\mathbf {Q}^{-1})$ can be replaced with approximations so that $\mathbf {Q}$ need not be inverted^25^. The running time of the approximation algorithm scales linearly with sample size. And the difference between the approximate CAR copula and the true copula can be made negligible except with respect to computational complexity.

I use the spam package^45^ for R to do sparse matrix computations. The chol and update functions of package spam perform the fast Cholesky decomposition and updating described above. And for smaller datasets the spam::chol2inv function can be used to invert $\mathbf {Q}$.

### Efficient computing for Gaussian processes

There are many approaches for speeding computing for Gaussian process models. I will briefly describe covariance tapering^46^, an approach that is appealing because it induces sparsity in an intuitive fashion and is effective for copula parameter estimation (and hence for the bootstrapping procedure for SGCRMs). When kriging (i.e., spatial interpolation), rather than regression, is of interest, other GP methods, such as the nearest-neighbor Gaussian process (NNGP)^47^, may perform better than covariance tapering. (The NNGP approach is, interestingly, reminiscent of the areal methods described above since the NNGP approach induces sparsity by defining a spatial process in terms of a well-chosen directed acyclic graph having vertices at the observed spatial locations).

In spatial covariance tapering, a spatial covariance (correlation) function is multiplied by a suitable positive definite function with compact support. The result is a spatial covariance (correlation) function that equals zero beyond a certain range. For example, if the spatial correlation function is the exponential correlation function $\exp \left( -3\,|\varvec{s}_i-\varvec{s}_{i'}|/\phi \right)$, which I used in Scenario 4 of my simulation study, Furrer et al.^46^ recommend the spherical tapering function. The spherical tapering function is given by

$$\begin{aligned} \tau _{ii'}&= \left\{ \left( 1-\frac{\Vert \varvec{s}_i-\varvec{s}_{i'}\Vert }{t}\right) _+\right\} ^2\left( 1+\frac{\Vert \varvec{s}_i-\varvec{s}_{i'}\Vert }{2t}\right) , \end{aligned}$$

where *t* is the taper length and $\bullet _+=\max \{0,\bullet \}$. Furrer et al.^46^ also considered tapers from the Wendland family, which prove useful for Matérn processes having smoothness $\nu >0.5$.

If $\mathbf {T}$ is the tapering matrix with $ii'$th entry $\tau _{ii'}$, the tapered correlation matrix is ${\varvec{\Omega }}_\text {tap} = {\varvec{\Omega }}\circ \mathbf {T}$. For a suitable taper length *t*, this matrix is sparse, and so fast sparse matrix algorithms can be used to compute $\vert {\varvec{\Omega }}_\text {tap}\vert$ and $\varvec{r}'{\varvec{\Omega }}_\text {tap}^{-1}\varvec{r}$. The resulting gain in computational efficiency can be dramatic.

## Analyses of Slovenia stomach cancer data

In this section, I revisit the Slovenian stomach cancer data. I applied four methods to the data: (1) ordinary Poisson regression with offset, (2) Poisson SGCRM with proper CAR copula as described in this paper, (3) Poisson SGCRM with Leroux copula, and (4) traditional Poisson SGLMM with proper CAR random effects. I used a bootstrap sample size of 4,000 for the SGCRM analyses. And I applied the SGLMM using Markov chain Monte Carlo for Bayesian inference, the customary approach for that model. I drew 1,000,000 posterior samples, which resulted in small Monte Carlo standard errors ($<0.003$)^48^.

For the ordinary GLM and the SGCRM, the transformed conditional mean is

$$\begin{aligned} \log \mathbb {E}(Y_i\mid x_i)=\log E_i+\beta _0+\beta _1x_i, \end{aligned}$$

where $Y_i$ is the observed count for the *i*th municipality, $E_i$ is the expected number of cases for the *i*th municipality, $\beta _0$ is an intercept term, and $\beta _1$ is the fixed effect for socioeconomic status, $x_i$. For the SGLMM this linear predictor was of course augmented by addition of a spatial random effect.

**Figure 4:**
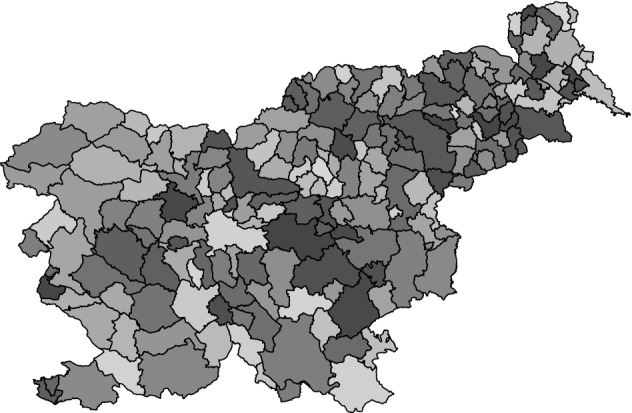
Standardized residuals for an ordinary GLM fit (Poisson regression with offset) to the Slovenia stomach cancer data. Darker gray means larger value. The residuals exhibit substantial, but short-range, positive dependence. Figure created using R version 4.1.2 (https://www.r-project.org).

The standardized residuals from the ordinary GLM fit are shown in Fig. 4. These residuals were used in the second stage of the SGCRM procedures to estimate the CAR copula parameter $\rho$ and Leroux copula parameter $\lambda$, and subsequently to incorporate spatial dependence in the bootstraps. We see that the standardized residuals clearly exhibit appreciable but fairly short-range spatial dependence. Specifically, we see regions of similarity, and said clusters are small relative to the extent of the map. To put it another way, we see small-scale spatial structure in these residuals. This visual assessment was corroborated by the estimate of $\rho$ ($\hat{\rho }=0.282$), which is shown in Table 4 along with the other results. (Recall that $\rho$ near 0 implies short-range dependence while $\rho$ near 1 implies long-range dependence.) The mild dependence in these residuals led to a confidence interval for $\beta _1$ that does not contain 0 and is just over 5% wider than the interval produced via the ordinary GLM. The Leroux model gave comparable results.

An astute reviewer pointed out that $\rho$ plays a complicated role in the joint distribution, as revealed by Wall^49^. Yet subsequent work by Assunção and Krainski^50^ established $\rho$ as a spatial range parameter. And so, values of $\rho$ that are close to 0 generally produce small-scale clustering in the data while values close to 1 produce large spatial clusters.

The SGLMM with CAR random effects produced a rather different estimate of $\varvec{\beta }$ and found no association between socioeconomic status and stomach cancer incidence. This is implausible given that a simple correlation analysis found a statistically significant association (Kendall’s $\hat{\tau }=-0.186$; p value < 0.001) between $Y_i/E_i$ and $x_i$. The non-significance of the SGLMM regression is due to the well-known phenomenon termed spatial confounding, i.e., collinearity between the spatial random effects and the fixed-effects predictors. Additionally, the CAR SGLMM found quite strong spatial dependence in the Slovenia data, yielding $\hat{\rho }=0.979$ (estimated posterior mode). This is puzzling and implausible given the agreement between the appearance of the residuals and the SGCRM’s small estimates of $\rho$ and $\lambda$. The SGLMM fit is difficult to defend while the SGCRM fit is plausible and satisfying.

**Table 4:** Results for analyses of the Slovenia stomach cancer data.

Approach	Intercept	Effect	Copula parameter	Running time
Ordinary GLM	$\hat{\beta }_0=0.156$	$\hat{\beta }_1=-0.137$; $\beta _1\in (-0.175, -0.098)$	–	$< 1$ s
SGCRM (CAR)	$\hat{\beta }_0=0.156$	$\hat{\beta }_1=-0.137$; $\beta _1\in (-0.177, -0.096)$	$\hat{\rho }=0.282$	18 s
SGCRM (Leroux)	$\hat{\beta }_0=0.156$	$\hat{\beta }_1=-0.137$; $\beta _1\in (-0.178, -0.094)$	$\hat{\lambda }=0.067$	11 s
SGLMM (CAR)	$\hat{\beta }_0=0.124$	$\hat{\beta }_1=-0.061$; $\beta _1\in (-0.142, 0.022)$	$\hat{\rho }=0.979$	1 h

The first row shows results for an ordinary Poisson regression with offset. The second row shows results for the two-stage SGCRM procedure, where the first stage employed an ordinary Poisson regression with offset. The third row shows results for the SGCRM procedure with Leroux copula. The fourth row shows results for an SGLMM fit such that the linear predictor for the Poisson regression with offset was augmented with proper CAR spatial random effects.

## Discussion

I believe the SGCRM, along with the two-stage inferential procedure developed in this paper, should be appealing to spatial modelers whose chief aim is to identify important explanatory variables when the response variable is spatially referenced. Because the SGCRM is a marginal model and thus cannot suffer from spatial confounding or other mixed-model pathologies, regression results from an SGCRM analysis have a clear and intuitive interpretation, namely, the same interpretation as for an ordinary regression model. And the bootstrap in the procedure’s second stage permits the standard errors for the estimated regression coefficients to be appropriately and efficiently adjusted in light of the extra-regression spatial dependence accommodated by the spatial Gaussian copula, the parameters of which can be estimated using standardized residuals from the first-stage fit.

I was inspired to write this paper by the excellent paper of Valle et al.^51^. They argue that one can get free of having to select the correct response family for count data (e.g., Poisson, negative binomial, zero-inflated Poisson, etc) by simply applying ordinal regression models to count data. Valle et al.^51^ show that an ordinal model fits count data better than the true model of the counts, owing to the ordinal model’s greater flexibility. Although Valle et al.^51^ did not consider spatially referenced counts, or, indeed, dependent counts more generally, it stands to reason that ordinal regression models hold the same promise for dependent counts as for independent counts. This suggests that the methodology I explored in this paper can be further unified for spatial count data.

Ungrouped binary spatial data present a unique challenge since the residuals from a Bernoulli GLM are typically worthless. In a future study I will carefully explore methods for regression analyses of spatially referenced ungrouped binary data, focusing especially on methods for continuous-domain spatial processes.

Another topic for future study is spatial smoothing, which I believe should more often be considered as distinct from spatial regression.

## Data Availability

The datasets analyzed during the current study are available from the corresponding author upon reasonable request.
